# Regulating the production of (*R*)-3-hydroxybutyrate in *Escherichia coli* by N or P limitation

**DOI:** 10.3389/fmicb.2015.00844

**Published:** 2015-08-19

**Authors:** Mónica Guevara-Martínez, Karin Sjöberg Gällnö, Gustav Sjöberg, Johan Jarmander, Mariel Perez-Zabaleta, Jorge Quillaguamán, Gen Larsson

**Affiliations:** ^1^Division of Industrial Biotechnology, School of Biotechnology, KTH Royal Institute of TechnologyStockholm, Sweden; ^2^Faculty of Science and Technology, Center of Biotechnology, Universidad Mayor de San SimónCochabamba, Bolivia

**Keywords:** *E. coli*, 3-hydroxybutyrate (3HB), polyhydroxybutyrate (PHB), fed-batch, phosphate, ammonium, limitation, depletion

## Abstract

The chiral compound (*R*)-3-hydroxybutyrate (3HB) is naturally produced by many wild type organisms as the monomer for polyhydroxybutyrate (PHB). Both compounds are commercially valuable and co-polymeric polyhydroxyalkanoates have been used e.g., in medical applications for skin grafting and as components in pharmaceuticals. In this paper we investigate cultivation strategies for production of 3HB in the previously described *E. coli* strain AF1000 pJBGT3RX. This strain produces extracellular 3HB by expression of two genes from the PHB pathway of *Halomonas boliviensis*. *H. boliviensis* is a newly isolated halophile that forms PHB as a storage compound during carbon excess and simultaneous limitation of another nutrient like nitrogen and phosphorous. We hypothesize that a similar approach can be used to control the flux from acetyl-CoA to 3HB also in *E. coli*; decreasing the flux to biomass and favoring the pathway to the product. We employed ammonium- or phosphate-limited fed-batch processes for comparison of the productivity at different nutrient limitation or starvation conditions. The feed rate was shown to affect the rate of glucose consumption, respiration, 3HB, and acetic acid production, although the proportions between them were more difficult to affect. The highest 3HB volumetric productivity, 1.5 g L^−1^ h^−1^, was seen for phosphate-limitation.

## Introduction

(*R*)-3-hydroxybutyrate (3HB) is a four-carbon alkanoate, which contains hydroxyl- and carboxyl-functional groups, as well as a chiral center. This chemical is thus proposed as an important building block for the synthesis of a range of valuable compounds (Seebach and Züger, 1982; Chen and Wu, 2005). For example, 3HB is an important precursor for the synthesis of carbapenem antibiotics (Chiba and Nakai, 1985; Chen and Wu, 2005) and di-*O*-methylelaiophylidene, a symmetrical macrodiolide antibiotic (Seebach et al., 1986). Dimers and trimers of 3HB have been considered as a means of providing energy to eukaryotic cells as precursors for ketone bodies (Tasaki et al., 1999). It has further gained particular attention as building block for synthesis of polyhydroxybutyrate (PHB) and its various copolymers; a family of microbially produced polyesters with multiple qualities and applications (Anderson and Dawes, 1990).

The commonly used production method for 3HB is hydrolysis of PHB that can be recovered from various types of wild type PHB producing organisms (Lee et al., 1999a,b; Chen and Wu, 2005). Here, the role model for both PHB and 3HB production is *Cupriavidus necator* (*Ralstonia eutropha*). An obvious drawback with this approach is the requirement to run two consecutive processes to arrive at the final product. This concept was first described for PHB production in wild type *Azohydromonas australica* (*Alcaligenes latus*) where hydrolysis with its natural depolymerase was performed (Lee et al., 1999a). An alternative process for 3HB production has been to use recombinant techniques to express the wild type genes in well-characterized and fast growing microorganisms such as *Escherichia coli*, a non-natural producer (Chen et al., 2013). Previously, cloning of the relevant genes from *C. necator* into *E. coli* was used for this purpose (Gao et al., 2002; Lee and Lee, 2003; Liu et al., 2007).

*Halomonas boliviensis* is a halophilic proteobacterium, which is able to produce PHB to high yield and with high productivity (81% of the cell dry weight and 1.1 g L^−1^ h^−1^, respectively) (Quillaguamán et al., 2008). One drawback with halophilic bacteria is however their requirement of atypical production conditions e.g., high salt concentrations that are unsuitable for use in common industrial bioreactors. Further the general lack of biological information about metabolism, physiology and genomics of wild type cells severely hampers the process of genetic manipulation and metabolic design. Due to the interesting genetic background of *H. boliviensis*, relevant also for 3HB production, we previously developed an *E. coli* strain with recombinant expression of a subset of the PHB producing genes (Jarmander et al., 2015).

**Figure 1:**
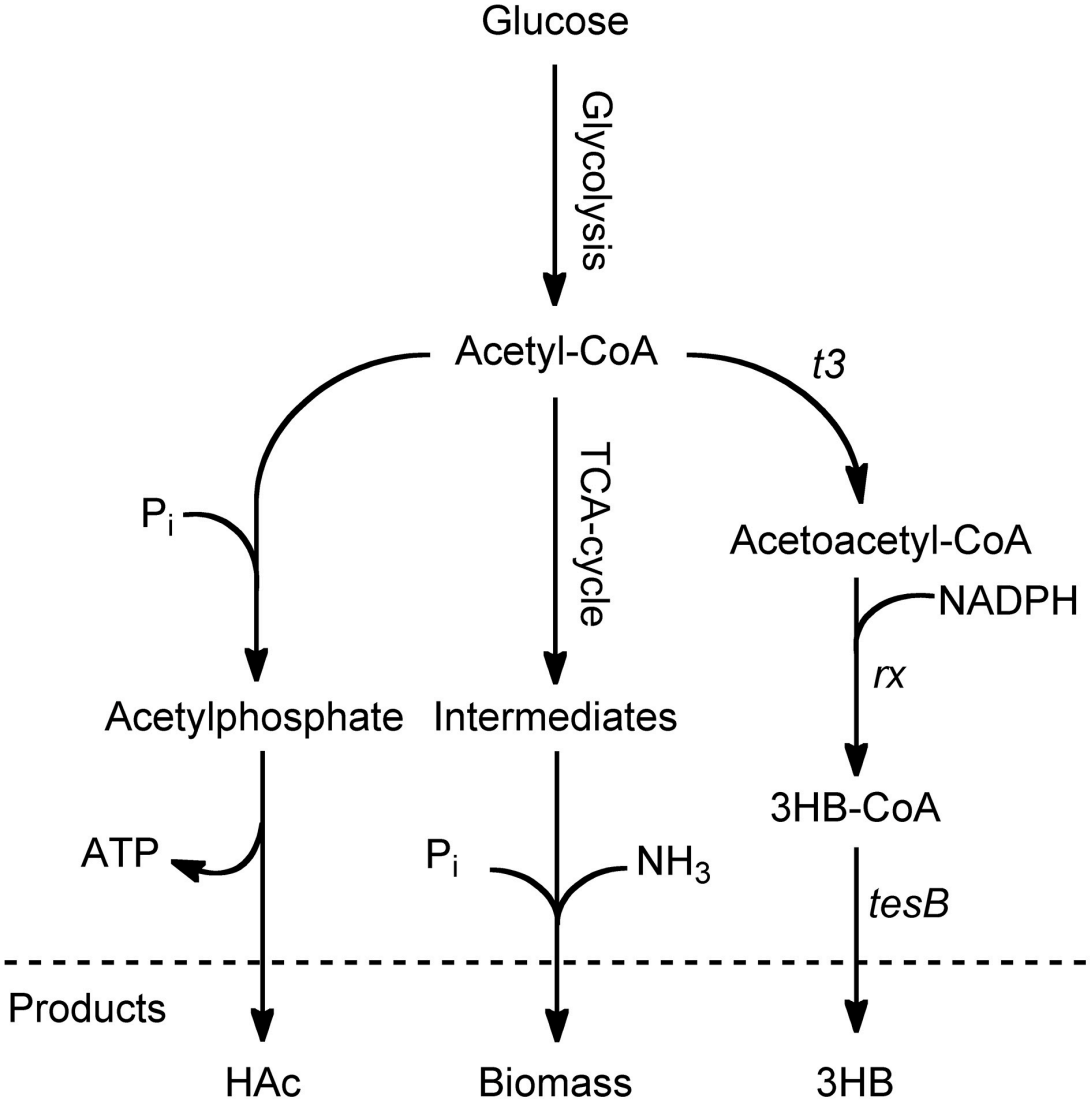
**Schematic overview of the main pathways surrounding acetyl-CoA in 3HB-producing *E. coli***. The production of 3HB from glucose begins with glycolysis, leading to the key intermediate acetyl-CoA. At this branch point, the carbon can be diverted to acetic acid (HAc) by the *pta/ack* pathway, it can be used for growth and energy through the TCA cycle, or it can be used for 3HB production through a pathway consisting of three steps. The first step is a condensation of two acetyl-CoA molecules to acetoacetyl-CoA, followed by reduction to 3-hydroxybutyryl-CoA. The final step is hydrolysis of 3-hydroxybutyryl-CoA to coenzyme A and 3HB, which could be catalyzed by endogenous thioesterase II (*tesB*).

A two-step metabolic route is generally used for the cellular production of 3HB-CoA with acetyl-CoA as precursor. The first reaction from acetyl-CoA to acetoacetyl-CoA is catalyzed by β-ketoacyl-CoA thiolase, followed by reduction to 3HB-CoA by acetoacetyl-CoA reductase, Figure 1 (Madison and Huisman, 1999). Since acetyl-CoA is one of the 12 main precursors for building of new cells, it constitutes an important branch point and is thus a substrate for many competing reactions (Neidhardt et al., 1990). It is the purpose of this paper to investigate a set of cultivation strategies to promote the flux of acetyl-CoA to 3HB-CoA. In *E. coli* 3HB-CoA is degraded into 3HB and CoASH. This degradation is likely catalyzed by the native thioesterase II (tesB), Figure 1, which in a previous study has been overexpressed to increase 3HB production in *E. coli* (Liu et al., 2007).

It is well-known that the metabolic flux to PHB in wild type cells is a result of a cellular strategy to promote the accumulation of storage carbon under conditions of starvation of a particular nutrient but under excess of a carbon source (Anderson and Dawes, 1990; Doi, 1990; Kim et al., 1994). This results in the accumulation of NADH to high levels, which is the inhibitor of citrate synthase in *E. coli* rather than ATP, as in eukaryotes (Weitzman, 1966). Inhibition of citrate synthase leads to a redistribution of the intracellular carbon flux from cell growth to polymer storage. Process design for both PHB and 3HB production, also in engineered cells, has thus generally been directed to mimic this principle, however only by keeping the sugar concentration at high level. This has been achieved by repeated batch procedures where large amounts of sugar have been added to cultivations, but where limitation or starvation was not clearly stated. The primary focus of such cultivation processes has thus been to maintain a high concentration of the carbon source and less emphasis has been put on the control and species of the limiting compound. A successful example of using a repeated batch for 3HB production, based on the genes from *C. necator* in *E. coli*, resulted in a maximum specific and volumetric productivity of 0.03 g g^−1^ h^−1^ and 0.51 g L^−1^ h^−1^, respectively, and with a final concentration of 12.2 g L^−1^ (Liu et al., 2007). In that study, the gene *tesB*, encoding thioesterase II, was co-expressed to transform 3HB-CoA to the free fatty acid, which was exported to the medium.

To improve productivity we hypothesize that the standard industrial concept fed-batch would constitute a more favorable cultivation strategy than, e.g., the repeated cycling of excess and starvation conditions. Fed-batch technology allows control of the cellular growth rate by continuous addition of a selected growth-limiting substance that should here not be the carbon source, which generally is used in industrial processing. The limitation makes it possible to avoid the severe effects on cell metabolism resulting from complete starvation and this might thus have a favorable effect on productivity. We initialized the experiments by collecting production capacity data from batch followed by starvation protocols, for reference. This was followed by fed-batch cultivation with glucose in excess, where either phosphorous or nitrogen was limiting and where productivity was studied at a set of relevant feed rates.

## Materials and methods

### Bacterial strain and plasmid

The bacterial strain used in this work was *E. coli* AF1000 (MC4100, relA1^+^) (Sandén et al., 2003). For 3HB production the plasmid pJBGT3RX (Jarmander et al., 2015) harboring two genes from *H. boliviensis; t3* (acetoacetyl-CoA thiolase) (WP_007111820) and *rx* (acetoacetyl-CoA reductase) (WP_007111780) was used. This plasmid was constructed from pKM1D, a pACYC184-derived low copy number plasmid with *ori* p15A, a lacUV5 promoter, a multiple cloning site, the lacIq repressor, and a chloramphenicol resistance gene.

### Cultivation procedure

#### Phosphate- and ammonium-depleted batch cultivations

*E. coli* cells from a glycerol stock stored at −80°C were inoculated to a sterile 5 L shake flask containing 500 mL of cultivation medium. The cells were cultivated overnight at 37°C and 180 rpm shaking, and were subsequently used to inoculate a sterile 15 L stirred tank bioreactor (STR) containing 10 L of cultivation medium to give a starting optical density at 600 nm of (OD_600_) of 0.1. The temperature was maintained at 37°C and the dissolved oxygen tension (DOT) was kept above 20% saturation by adjusting the air flow and stirring speed. Antifoam was added when required. Production of 3HB was induced at an OD_600_ of 0.2 by addition of 200 μM isopropyl β-D-1 thiogalactopyranoside (IPTG). Samples for OD_600_, cell dry weight (CDW), glucose, 3HB, acetic acid (HAc), and ammonium or phosphate were withdrawn regularly during cultivations. An approximate sample volume of 17 ml was taken out at each sampling point.

#### Phosphate- and ammonium-limited fed-batch cultivations

*E. coli* cells from a glycerol stock stored at −80°C were inoculated directly into a sterile 15 L STR containing 10 L cultivation medium. The inoculation volume was adjusted to give an OD of 0.1 in the reactor after 15 h of cultivation. The cultivation temperature was 37°C and the DOT was kept above 20% saturation by adjusting the air flow and stirring speed. Antifoam was added when required. The cells were induced at OD_600_ = 0.2 with 200 μM IPTG. When the batch phase was over, indicated by an increase in DOT, feeding of the limiting substrate (phosphate or ammonium) was initiated. The feed was divided into two phases; an exponential phase and a linearly increasing phase. The exponential phase is described by Equation (1), where the feed was set to give a specific growth rate (μ) of approximately 0.35 h^−1^.

(1) $$\begin{array}{l} F=F_{0}e^{\mu t} \end{array}$$

The exponential feed was switched into a linearly increasing feed when the OD_600_ reached a value of 40, and is described by Equation (2).

(2) $$\begin{array}{l} F=F_{0}+0.1t \end{array}$$

*F*_0_ is the initial feed rate and is defined below, where x is the CDW at feed start, *V* is the volume in the reactor, *S* is the concentration of ammonium or phosphatein the feed and *Y*_*xs*_ is the yield of cells over ammonium or phosphate.

(3) $$\begin{array}{l} F_{0}=\frac{\mu xV}{SY_{xs}} \end{array}$$

Glucose was fed with the same profile as the limiting substrate to keep carbon in excess, if the glucose concentration decreased below 5 g L^−1^, a batch of glucose was added to the reactor. 180 ml of 500 g ml^−1^ glucose was added at 5 h and 120 ml of 500 g ml^−1^ glucose was added at 11.5 h for the phosphate-limited fed batch. In the ammonium-limited fed-batch no extra glucose was added. Samples for OD_600_, CDW, glucose, 3HB, HAc, and ammonium or phosphate were withdrawn regularly during cultivation. An approximate sample volume of 17 ml was taken out at each sampling point.

### Cultivation medium

The cultivation medium used was based on a minimal salt medium consisting of 15 g L^−1^ glucose (sterilized and added separately), 5 g L^−1^ (NH_4_)_2_SO_4_, 1.6 g L^−1^ KH_2_PO_4_, 0.7 g L^−1^ Na_3_C_6_H_5_O_7_•2H_2_O, 6.6 g L^−1^ Na_2_HPO_4_•2H_2_O, 50 mg L^−1^ chloramphenicol, and 50 μL L^−1^ antifoam. Addition of 1 mL L^−1^ of sterile 1 M MgSO_4_ and 1 mL L^−1^ sterile trace element stock solution (0.5 g L^−1^ CaCl_2_•2H_2_O, 16.7 g L^−1^ FeCl_3_•6H_2_O, 0.18 g L^−1^ ZnSO_4_•7H_2_O, 0.16 g L^−1^ CuSO_4_•5H_2_O, 0.15 g L^−1^ MnSO_4_•4H_2_O, 0.18 g L^−1^ CoCl_2_•6H_2_O, 20.1 g L^−1^ Na-EDTA) was made after sterilization of the minimal medium. For fed-batch cultivations, 1 mL L^−1^ of sterile 1 M MgSO_4_ and 1 mL L^−1^ sterile trace element stock solution was added for every increase of 10 in OD_600_.

In the phosphate-depleted cultivation, Na_2_HPO_4_•2H_2_O was omitted and the level of KH_2_PO_4_ was adjusted to 0.16 g L^−1^. In the ammonium-depleted cultivation, the level of ammonium sulfate was adjusted to 1 g L^−1^. For the phosphate-limited fed-batch, Na_2_HPO_4_•2H_2_O was omitted and the initial concentration of KH_2_PO_4_ was 0.3 g L^−1^. In the ammonium-limited fed-batch, the initial concentration of ammonium sulfate was 2 g L^−1^. For the fed-batch cultivations, a feed-solution consisting of 500 g kg^−1^ of glucose was fed to maintain glucose in excess. For feeding of phosphate or ammonium, feed solutions containing 60 g kg^−1^ KH_2_PO_4_or 250 g kg^−1^ (NH_4_)_2_SO_4_ were used. 25% (w/v) NH_4_OH was used for pH-titration in all cultivations except for the ammonium-depleted and -limited cultivations, where 3 M NaOH was used.

### Analyses

#### Cell growth

Samples for OD_600_ were diluted in 0.9% w/v NaCl in duplicate to an approximate OD_600_ of 0.1 prior to measurement in a spectrophotometer at 600 nm (Genesys 20, Thermo scientific). Cell growth was also monitored with CDW, where 5 mL of cell culture was withdrawn into pre-weighed, dry glass tubes and subsequently centrifuged at 2000 *g* for 10 min in a tabletop centrifuge (CompactStar CS4, VWR). Sampling was done in triplicates. The supernatant was thereafter removed and the cell pellets were dried overnight at 105°C. The dry pellets were allowed to cool to ambient temperature in a desiccator and were subsequently weighed.

#### Glucose, ammonium, and phosphate analysis

A sample of 2 mL cell culture was withdrawn rapidly into a pre-weighed tube containing 2 mL of cold (4°C) perchloric acid at a concentration of 0.13 M to stop metabolism (Larsson and Törnkvist, 1996). The tube was centrifuged at 2000 *g* for 10 min and 3.5 mL of the supernatant was neutralized with 75 μL saturated (500 g L^−1^) potassium carbonate. The sample was thereafter put on ice for 15 min to allow for salts to precipitate. The sample was subsequently centrifuged at 2000 *g* for 5 min and the supernatant was stored at −20°C until analysis. The substrate concentrations were determined using commercially available enzymatic kits (Boehringer Mannheim Cat No. 716251, Megazyme Ammonia Kit Cat No. K-AMIAR, abcam Phosphate Assay Kit (Colorimetric) Cat No. ab65622).

#### Acetic acid and 3-hydroxybutyrate analysis

The supernatant from the CDW samples was filtered (0.2 μm, VWR collection) and stored at −20°C until analysis. The concentration of HAc and 3HB was determined using commercially available enzymatic kits (R-BIOPHARM acetic acid Kit Art. No. 10148261035, Megazyme D-3-hydroxybutyric acid assay kit Cat No. K-HDBA).

### Calculation of rates

The values for cell mass were fitted with a function, (4), in the appropriate interval by a least square regression. The product, P, was also fitted with a function, (5), in a similar fashion. The volumetric rates, (6), were defined as the derivative of (5).

(4) $$\begin{array}{l} f\left(t\right) \end{array}$$

(5) $$\begin{array}{l} g\left(t\right) \end{array}$$

(6) $$\begin{array}{l} g^{\prime }\left(t\right)=r_{P} \end{array}$$

The specific rates, (7), were defined as the volumetric rate divided by the function for cell mass (4).

(7) $$\begin{array}{l} \frac{g^{\prime }\left(t\right)}{f\left(t\right)}=q_{P} \end{array}$$

In the special case of cell mass; q_*P*_ = q_*CDW*_ = μ.

## Results

### Repeated batch cultivations

Common cultivation protocols for 3HB production in *E. coli* have been characterized by the aim of keeping the concentration of carbon source high by the use of the repeated batch concept. In these cultivations, the batch phase was prolonged either by continuous or intermittent addition of sugar, before the cells entered the stationary phase. In this way, Liu et al. intermittently added a combination of glucose, ammonium, and magnesium sulfate to the cultivation (Liu et al., 2007) while Gao et al. added glucose, yeast extract, and magnesium sulfate by continuous feeding (Gao et al., 2002). The glucose concentration hereby stayed above 10 g L^−1^. Neither of these cultivations seems to be designed for nutrient-starvation, which generally constitutes the requirement accumulation of storage compounds like PHB in wild type cells, as previously discussed.

As a starting point, we investigated 3HB production under nutrient excess with recombinant *E. coli* cells containing a set of the relevant *H. boliviensis* genes. This was done in a simple batch cultivation, to confirm that sugar excess by itself, is not enough to produce storage-type products and the yield under these conditions was indeed very low, i.e., 0.06 g g^−1^ (Jarmander et al., 2015). Our hypothesis is that to obtain a high 3HB yield, the flux to this product has to be promoted by inhibition of citrate synthase and thereby growth, as in polymer storage production and this requires limitation or starvation of a specific compound other than sugar.

**Figure 2:**
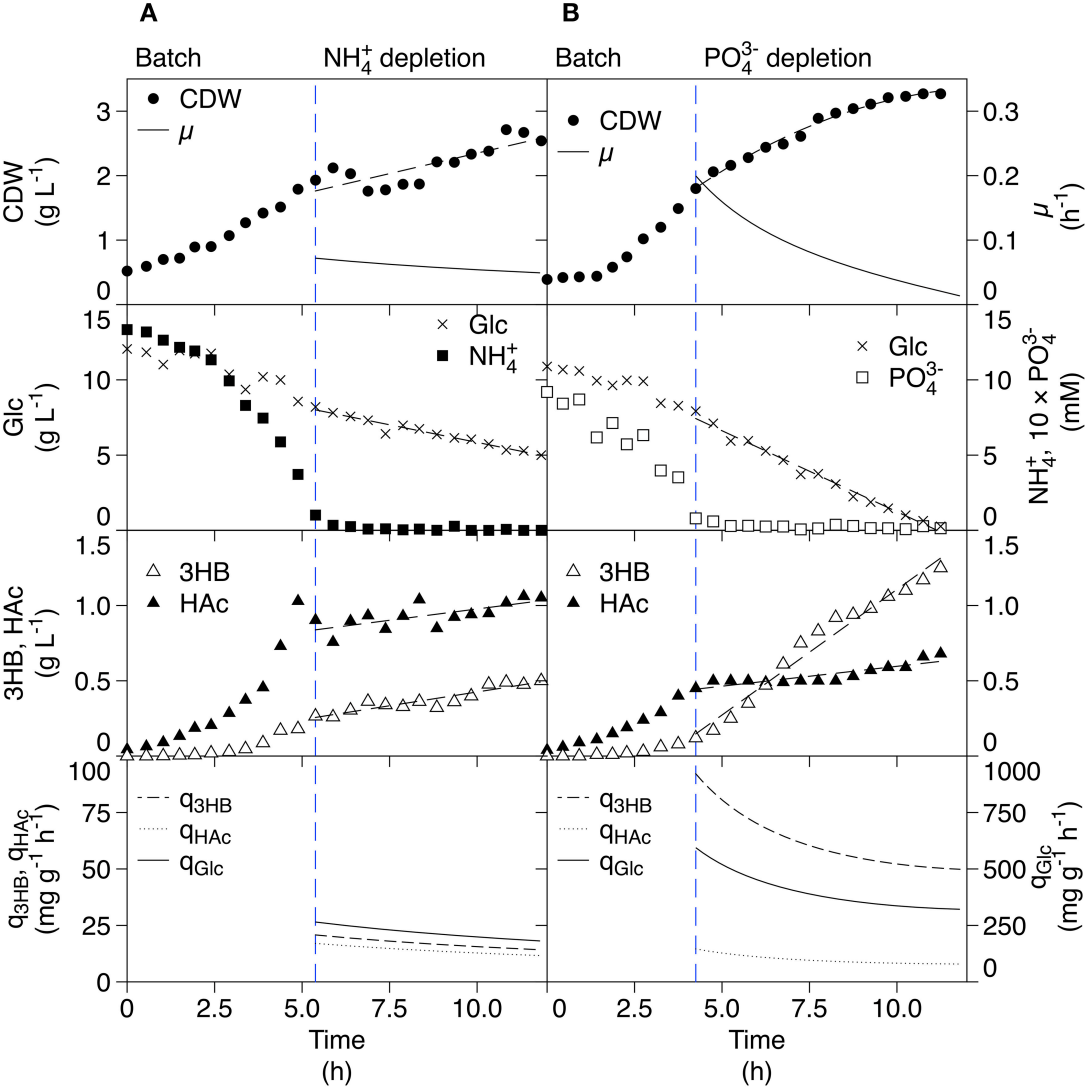
**Reference batch cultivations of AF1000 pJBGT3RX with depletion of either ammonium (A) or phosphate (B) for production of 3HB**. The measured parameters were: cell dry weight (CDW, filled circles), glucose (Glc, crosses), ammonium (NH${}_{4}^{+}$, filled squares), phosphate (PO${}_{4}^{3-}$, open squares), acetic acid (HAc, filled triangles), and (*R*)-3-hydroxybutyrate (3HB, open triangles). Specific rates (μ, q_3HB_, q_Hac_, and q_Glc_) are represented as functions obtained from least square fits of the data. All parameters except ammonium, phosphate and CDW **(B)** were fit with first order polynomials. CDW **(B)** was fit with a second order polynomial.

Former 3HB production was done with high carbon levels followed by nutrient-starvation. As a reference to that procedure, batch cultivations followed by a starvation phase were performed. The batches were divided into two phases; a batch phase with all nutrients in excess, and a depletion phase that started after complete consumption (depletion) of ammonium or phosphate. In Figure 2A, the ammonium-depleted cultivation is shown. After depletion of ammonium, uptake of glucose is still present and the cell dry weight (CDW) increases slightly in a linear fashion. Depletion of ammonium results thus in that no conventional stationary phase, as for glucose-starvation, is observed. This is in line with what is seen for *Klebsiella pneumonia* after ammonium-depletion, where the increase in cell mass is described by accumulation of carbon storage compounds rather than actual cell growth (Wanner and Egli, 1990). The nitrogen in this case is thought to come from degradation of intracellular nitrogen-rich compounds, leading to reduced cellular protein content.

Product formation could be observed as soluble 3HB in the medium since 3HB-CoA was hydrolyzed and 3HB excreted by *E. coli*-specific enzymes, possibly thioesterase II which was previously shown to perform this cleavage (Liu et al., 2007). Production of 3HB was observed for both the batch and the ammonium-depleted phase; in the batch phase, the increase in 3HB concentration followed an exponential pattern whilst it increased linearly in the depleted phase. The specific productivity of 3HB (q_3HB_)in the depleted phase was almost constant over a time span of 7 h with a mean value of 17 mg g^−1^ h^−1^. The yield of 3HB with respect to CDW was 0.57 g g^−1^ as compared to 0.06 g g^−1^ for conditions of nutrient excess.

In batch processes, *E. coli* commonly produces acetic acid (HAc) as an overflow metabolite. This is an immediate effect of high sugar uptake and glycolytic flux. The effect is confirmed by the HAc production seen in the batch phase. However, HAc production continued in the ammonium-depleted phase where it followed the same pattern as 3HB production, rendering a specific HAc productivity (q_HAc_) close to the one of 3HB. For 3HB production, it is important to minimize HAc production since it competes with 3HB for the common substrate acetyl-CoA, Figure 1.

The phosphate-depleted cultivation is shown in Figure 2B and also here, glucose is continuously assimilated in the starvation phase. The CDW increased to a much greater extent than for ammonium-depletion, this can be explained by the differences in metabolism of phosphate and ammonium. The cells have intracellular reserves of phosphate in form of polyphosphates, rRNA, and phospholipids which can be utilized when phosphate is scarce, while there is no pool of readily available nitrogen (Wanner and Egli, 1990). The increase in CDW seen during external phosphate starvation is thus overcome by an internal supply due to macromolecular hydrolysis. The production of 3HB was further considerably higher compared to ammonium-starvation, with a mean productivity of 65 mg g^−1^ h^−1^ and a yield of 0.89 g g^−1^ which was accompanied by a substantially lower HAc production rate that seems here to be uncoupled from 3HB production.

### Fed-batch cultivations

The fed-batch technique is commonly used in biotechnology industry and some of its many benefits are that high cell densities can be accumulated without restrictions in oxygen or heat transfer. Another advantage is the possibility to control specific growth rate (μ) by feeding of a limiting substrate, most often the carbon source, resulting in reactor concentrations close to the K_*s*_-value of the chosen substrate. Using carbon as the limiting substrate is of course excluded from our purpose since a high carbon flux is desired. Alternatively, the ammonium or phosphate concentration can be restricted so that a high carbon flux is maintained. The strategy was thus to feed ammonium or phosphate at a rate that was designed to force excess carbon toward production of 3HB to avoid starvation.

To test a range of feed profiles, while still keeping to common fed-batch protocols, the cultivation was divided into three phases; (1) a batch phase, (2) an exponential feed phase, and (3) a linear feed phase. The batch phase was designed to end either due to ammonium or to phosphate depletion. After the batch phase, the two feed phases followed in a successive mode, where both glucose and the limiting component were fed simultaneously, which was necessary to maintain carbon excess. The two feed phases are radically different; exponential feed allows the cells to grow exponentially at constant growth rate while linear feed leads to a successively reduced growth rate due to less substrate availability per cell. This allowed investigation of increasing substrate-limitation. However, at a point in the linear feed phase, the requirements of sugar increased radically and the automatic glucose feed was not enough to maintain carbon in excess. To avoid carbon-limitation, glucose was added batch-wise in addition to the feed for the phosphate-limited case.

**Figure 3:**
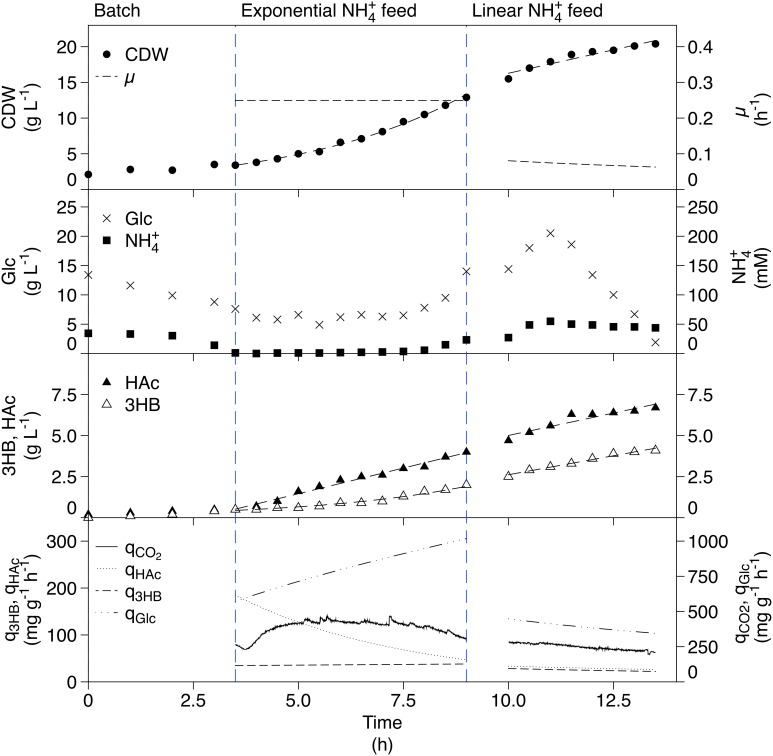
**Ammonium-limited fed-batch cultivation of AF1000 pJBGT3RX for production of 3HB**. The measured parameters were: cell dry weight (CDW, filled circles), glucose (Glc, crosses), ammonium (NH${}_{4}^{+}$, filled squares), acetic acid (HAc, filled triangles) and (*R*)-3-hydroxybutyrate (3HB, open triangles). Specific rates (μ, q_3HB_, q_Hac_, q_Glc_, and q_CO2_) are represented as functions obtained from least square fits of the data and online measurements. CDW and 3HB have been fit with an exponential function in the exponential feed phase and a first order polynomial in the linear feed phase. HAc was fit with a first order polynomial in both phases.

The ammonium-limited fed-batch cultivation is shown in Figure 3. The CDW increased in the feed phases as expected. This resulted in a constant μ of 0.26 h^−1^ in the exponential phase, while in the linear feed phase, the CDW increased linearly resulting in a decreasing specific growth rate. Glucose was in excess in all phases while the ammonium concentration was limiting. An unexpected accumulation of ammonium was detected at the end of the exponential feed phase but despite this, growth still seems to be limited. Production of 3HB was seen in all phases; exponential production in the exponential feed phase and linear production in the linear feed phase. The specific 3HB productivity did not change markedly between the two phases. The final concentration of 3HB was 4.1 g L^−1^. HAc was produced in both the exponential and linear feed phases. For the linear feed phase, q_HAc_ followed q_3HB_ closely, giving a final HAc concentration of 6.7 g L^−1^. HAc production is not expected when growth is restricted in fed-batch cultivations but in this cultivation setup, where glucose is in excess, the scenario is different.

**Figure 4:**
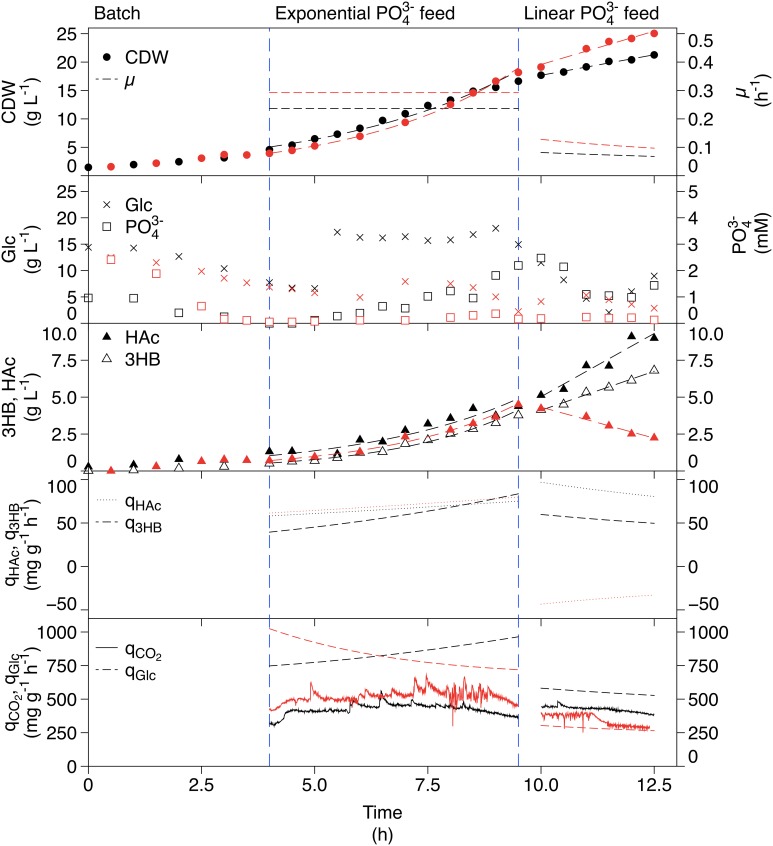
**Phosphate-limited fed-batch cultivation of AF1000 pJBGT3RX for production of 3HB (black) compared to a non-induced replicate cultivation without 3HB production (red)**. The measured parameters were: cell dry weight (CDW, filled circles), glucose (Glc, crosses), phosphate (PO${}_{4}^{3-}$, open squares), acetic acid (HAc, filled triangles), and (*R*)-3-hydroxybutyrate (3HB, open triangles). Specific rates (μ, q_3HB_, q_Hac_, q_Glc_, and q_CO2_) are represented as functions obtained from least square fits of the data and online measurements. CDW, HAc, and 3HB have been fit with an exponential function in the exponential feed phase and a first order polynomial in the linear feed phase.

The phosphate-limited fed-batch cultivation is shown in Figure 4. The CDW increased exponentially resulting in a μ of 0.25 h^−1^ in the exponential feed phase. In the linear feed phase, the CDW increased linearly, which led to a slow decrease in μ in the same manner as for the ammonium-limitation. Glucose was kept in excess even though the concentration dropped to 2 g L^−1^ in the linear feed phase. Phosphate accumulated in the end of the exponential feed phase but it decreased again during the linear feed phase. Production of 3HB correlated well with the feed profile. The specific production rate was however considerably higher than that in the ammonium-limited fed-batch cultivation, giving a final 3HB concentration of 6.8 g L^−1^. HAc was produced in all phases, also at a rate corresponding to the feed rate, and reached a final concentration of 9.0 g L^−1^.

The phosphate-limited cultivation had the highest specific productivity and final concentration of 3HB, which makes it interesting for optimization studies. However, a considerable amount of carbon is directed to HAc production, which is undesirable for productivity reasons. The question was then if HAc was produced due to phosphate-limitation, or whether the increased production was due to the increased 3HB production. This was investigated in a replicate phosphate-limited fed-batch where the gene expression was not induced. The data from this cultivation is included in red in Figure 4. The HAc production was similar to the cultivation with production in the exponential feed phase but in the linear feed phase there was a large difference; while HAc was produced during 3HB production, it was consumed in the non-induced case, even though glucose was in excess. This means that the production of 3HB is strictly correlated to production of HAc. It should also be observed that the consumption of glucose was very different between the two cultivations, being approximately two times as high during 3HB production. The specific carbon dioxide production rate (q_CO2_) was in the same range for both the induced and non-induced cultivations indicating that the increased glucose consumption was not used for respiration i.e., it was used for 3HB and HAc production.

## Discussion

### 3HB production in nutrient-depleted batch conditions

The reference cultivations for 3HB production in the ammonium- and phosphate-depleted phases of batch cultivations have been performed as a benchmark for the most common metabolite production processes. The first observation that can be made with regard to production is that the cells continue to produce 3HB despite lacking ammonium and phosphate, much like wild type PHB producers. Also, the cells continue to grow in the depleted phase, as expected (Wanner and Egli, 1990). Interestingly, the rate of 3HB production is much higher in the phosphate-depleted phase than in the ammonium-depleted phase, although there is a decrease in q_3HB_ after some time of phosphate depletion. Here, q_3HB_ remains rather high, at above 46 mg g^−1^ h^−1^, compared to the ammonium-depleted case, where q_3HB_ never exceeds 21 mg g^−1^ h^−1^. In conclusion, the positive effect of complete nutrient depletion is a high yield of 3HB per CDW, but this is due to that the cells stop to grow almost completely, which of course is an unfavorable situation as there is a need for high volumetric productivity.

### The benefits of fed-batch cultivation

To evaluate 3HB production at limiting but not completely depleted phosphate and ammonium conditions, fed-batch cultivations were performed. It could be shown that the volumetric productivity, r_3HB_, increased in both the ammonium- and the phosphate-limited cases when going from batch to fed-batch conditions (Table 1). The highest volumetric productivity observed was at the end of the exponential feed phase in the phosphate-limited fed-batch, where r_3HB_ reached 1500 mg L^−1^ h^−1^. The main factor at work here is the increase in CDW during the feed phases compared to the depleted phase, where μ is very low and approaches zero. Increase in r_3HB_ due to increasing cell mass has also been observed for the repeated batch experiments performed previously (Gao et al., 2002; Liu et al., 2007). However, use of a feed to limit nutrient concentration is the more common industrial protocol, since it allows for automation and better growth control of the cultivation.

**Table 1:** **3HB production for different cultivation strategies; depletion, exponential feed, and linear feed**.

	**q_3HB_ (mg g^−1^ h^−1^)**	**r_3HB_ (mg L^−1^ h^−1^)**	**Final 3HB concentration (g L/^−1^)**	**CDW (g L/^−1^)**	**Y/_3HB∕CDW_ (g g/^−1^)**
**AMMONIUM**					
Depletion	14–21	37	0.50	2.54	0.57
Exponential feed	35–38	120–510	2.00	12.93	0.16
Linear feed	22–28	460	4.10	20.40	0.20
**PHOSPHATE**					
Depletion	50–92	170	1.25	3.27	0.89
Exponential feed	39–84	200–1500	2.85	14.87	0.23
Linear feed	50–60	1000	6.81	21.27	0.32

The values given in the table are calculated over the respective phases and do not include the batch phases. Values given in a range represent start and end values of the corresponding function.

In addition to increased r_3HB_, the fed-batch cultivation resulted in a higher q_3HB_ for the ammonium-limited case (Table 1). A higher μ correlated with a higher specific productivity at the levels tested (depletion, exponential feed μ ≈ 0.25 and linear feed μ ≈ 0.1). In the phosphate-limited fed-batch the specific glucose uptake (q_Glu_) was considerably higher than in the ammonium-limited fed-batch leading to a higher glycolytic flux and a higher q_3HB_, giving phosphate-limitation a distinct advantage as a production strategy.

Earlier studies on *E. coli* PPA652ara grown on lignocellulosic sugars in ammonium-limited fed-batch showed that it is possible to produce 3HB at comparable specific productivities as in AF1000 pJBGT3RX grown on glucose (Jarmander et al., 2015). Our results indicate that the productivity for PPA652ara can be increased if phosphate-limited fed-batch was to be used as production strategy.

### Comparison of phosphate and ammonium depletion

The glucose uptake rate is considerably higher in the phosphate- compared to the ammonium-limited case, which leads to a higher q_3HB_. The differences between phosphate and ammonium depletion can be attributed to the way that microorganisms respond to the respective limitations. In general, we have observed a higher metabolic activity for the phosphate-limited cells than ammonium-limited cells (Figures 3, 4, μ, q_HAc_, and q_CO2_), which can be explained by the presence of an internal phosphate storage in the form of polyphosphates, phospholipids, and RNA. These compounds can be capitalized on and used for further growth, until the supply runs out. In the ammonium-limited case on the other hand, the comparatively low availability of internal nitrogen means that growth can only continue for the already initiated replication forks as formerly suggested, meaning that the cells at most could grow an extra generation (Wanner and Egli, 1990).

### 3HB flux distribution under phosphate-limitation

In order to investigate if the increased metabolic rate and HAc production is caused by a combination of 3HB production and phosphate-limitation, or due to limitation alone, a reference fed-batch without 3HB production was performed. Here, we could observe that q_CO2_ was almost identical between the two phosphate-limited cultivations, but considerably higher than for ammonium-limitation. The yield of CO_2_ produced per glucose consumed however remained constant, meaning that no extra carbon was directed to respiration. The specific growth rate is controlled by the feed rate of phosphate and this affects the specific glucose uptake rate, giving a higher μ in the exponential compared to the linear phase. The flux to acetic acid co-varies with the flux to 3HB, which means that a considerable amount of carbon also ends up as HAc. The HAc production in the linear feed phase is coupled to 3HB production, and not phosphate-limitation, since HAc is consumed in this phase for the non-induced phosphate cultivation. In the non-induced case, the glucose consumption rate is considerably lower than in the 3HB production case, which leads to a lack of carbon in the cell and allows simultaneous uptake of HAc and glucose. In the case of 3HB production, there is an increased need for energy, which can be supplied by HAc production (Figure 1).

The only case where HAc production is decoupled from 3HB production is the case of phosphate-starvation. Here, the ratio of 3HB production to HAc production is much higher than in any other cultivation. This could be due to the requirement for phosphate in the pathway for HAc production (Figure 1).

## Author contributions

MG performed the majority of the experimental work and contributed to the manuscript. KG contributed to the experimental work and wrote the manuscript. GS and JJ contributed to the experimental work and the manuscript. MP contributed to the experimental work and revised the manuscript. JQ and GL were responsible for the original concept and supervised the work. JQ revised the manuscript. GL contributed to the manuscript. All authors have read and approved the manuscript.

## Funding

The Swedish Research Council Formas (211-2013-70) and The Swedish International Development Agency (SIDA).

### Conflict of interest statement

The authors declare that the research was conducted in the absence of any commercial or financial relationships that could be construed as a potential conflict of interest.
